# Mental disorder symptoms and diagnoses are differently associated with labour market attachment and registered income until midlife: The Northern Finland Birth Cohort 1966

**DOI:** 10.1177/00207640241299384

**Published:** 2024-11-27

**Authors:** Tuomas Majuri, Sanna Huikari, Erika Jääskeläinen, Leena Ala-Mursula, Ina Rissanen, Marko Korhonen

**Affiliations:** 1 Research Unit of Population Health, University of Oulu, Finland; 2 Medical Research Center Oulu, Oulu University Hospital and University of Oulu, Finland; 3 Department of Psychiatry, Oulu University Hospital, Finland; 4 Department of Economics, Accounting and Finance, University of Oulu, Finland; 5 Julius Center for Health Sciences and Primary Care, University Medical Center Utrecht and Utrecht University, The Netherlands; 6 Department of General Practice, Amsterdam UMC and University of Amsterdam, The Netherlands

**Keywords:** Mental disorder, income, employment, labour market attachment, follow-up, cohort study

## Abstract

**Background:**

Both the symptoms and diagnoses of mental health disorders affect individuals’ occupational status and income. However, studies that compare the impact of differences between symptoms and diagnoses on occupational outcomes are lacking.

**Aims:**

This study aimed to compare labour market attachment and income until midlife between individuals with different histories of mental disorder symptoms and diagnoses.

**Method:**

Utilizing the Northern Finland Birth Cohort 1966 with linkages to national registers and self-reported mental disorder symptoms at the age of 31, we compared labour market attachment and income until midlife among individuals with neither mental disorder symptoms nor a diagnosis (reference group), symptomatic undiagnosed mental disorder, asymptomatic diagnosed mental disorder, and with symptomatic diagnosed mental disorder by using cross-tabulations and regression analyses. We stratified our analysis by sex.

**Results:**

Compared to the reference group, males but not females with symptomatic undiagnosed mental disorder had an increased risk for poor labour market attachment, with Odds Ratios (95% Confidence intervals) 2.26 [1.41, 3.63] and 0.87 [0.63, 1.19], respectively. The analogous risk was heightened for both males and females with asymptomatic diagnosed mental disorders or symptomatic diagnosed mental disorders. Regarding income, having mental disorder symptoms, a diagnosis, or both was associated with lower earnings irrespective of sex.

**Conclusions:**

Mental disorder symptoms and diagnoses are differently associated with labour market attachment and income. Our findings suggest that interventions should be offered not only based on diagnoses but also based on symptoms as they may serve as predictors of future challenges related to work careers.

## Introduction

Mental disorders are the largest cause of work disability in many nations (OECD, 2010). Common mental disorders have been linked with poor socioeconomic outcomes in terms of income and occupational status in numerous studies (Hakulinen et al., 2019, 2020).

Self-reported mental disorder symptoms and clinician-based mental disorder diagnoses are common measures to describe poor mental health. However, discrepancies exist between symptoms and mental disorder diagnoses. Individuals with current symptoms may not meet the diagnostic criteria for a mental disorder (Ortiz-Lobo et al., 2011) or may remain undiagnosed (Sacco et al., 2024). Conversely, individuals with a mental disorder diagnosis may be in remission (Catalan et al., 2021; Whiteford et al., 2013), recovered (Catalan et al., 2021; Roach et al., 2023), misdiagnosed (Ayano et al., 2021) or overdiagnosed (Davis et al., 2016; Thombs et al., 2019) and currently without apparent symptoms.

Both mental disorder symptoms (Hiilamo et al., 2019; Pedersen et al., 2016) and diagnoses (Hakulinen et al., 2019, 2020; Majuri et al., 2023) have an impact on individuals’ occupational outcomes. Symptom experience is the factor that affects people’s everyday functioning and work ability. Conversely, a mental disorder diagnosis is needed to be able to receive sickness allowances and disability benefits that inherently affect labour market attachment. Most of the studies have explored the associations between either mental disorder symptoms and occupational outcomes (Hiilamo et al., 2019; Pedersen et al., 2016) or diagnoses and outcomes (Hakulinen et al., 2019, 2020; Majuri et al., 2023), often overlooking each other. Few studies have specifically focused on exploring the gap between symptoms and diagnoses (Fisher et al., 2021; Waserstein et al., 2019; Young et al., 2020), including individuals with undiagnosed and subclinical mental disorders, those in remission or who have recovered and those with other discrepancies between symptoms and mental disorder diagnoses. Studies comparing the impact of differences between mental disorder symptoms and diagnoses on occupational outcomes are lacking.

Biological, psychological, and social differences between males and females lead to distinct mental health challenges and stressors related to work-life balance (Koenig & Eagly, 2014). Sex differences in mental health (Campbell et al., 2021; Yu, 2018), employment opportunities (International Labour Office, 2017), and the work-family life courses of general population samples (McMunn et al., 2015) have been reported. Due to sex inequalities leading to unequal impact on employment and earnings trajectories, sex-specific de-standardization of trajectories has been suggested (Widmer & Ritschard, 2009). Ignoring these differences could obscure important findings, lead to biased results, and limit the applicability of conclusions drawn from the analysis. We stratified our analysis by sex to ensure that data interpretation and subsequent interventions are accurate, equitable, and appropriately tailored to different populations. By combining national-level register-based data on mental disorder diagnoses with self-reported mental disorder symptoms at the age of 31, the study aimed to compare labour market attachment and income until midlife between individuals with different histories of mental disorder symptoms and diagnoses. The data included register-based data linked to the Northern Finland Birth Cohort 1966 (NFBC1966) with longitudinal survey data on 30 years of working life.

## Methods

### Sample

The study was based on the NFBC1966 (University of Oulu, 1966), which is an unselected, prospective, general population sample comprising information on 12,058 live-born children in the provinces of Oulu and Lapland, with expected dates of birth in 1966. The cohort members have been followed up with data collection at different ages with four main follow-up surveys occurring at the age of one, 14, 31, and 46 years, including linkages with national register data (Online Resource 1) (Nordström et al., 2022). Cohort participants have the option to continually update their consent and the number of study subjects can thus vary depending on the time of the study.

We identified cohort members responding to the Hopkins Symptom Checklist-25 (HSCL-25) at the age of 31, to the questionnaire on work, economy and resources, including annual employment roles between ages 16 and 45 at the age of 46 years, and allowing their data to be used in this research. These individuals comprised the final sample of the study (n = 5,831).

### Measures

#### Self-reported Mental Disorder Symptoms

The HSCL-25 is a validated questionnaire, used to identify mental disorders in primary care (Sandanger et al., 1998; Veijola et al., 2003). The HSCL-25 includes 25 questions about the presence and intensity of depression and anxiety symptoms during the previous week. The intensity of each symptom is assessed on a four-point scale from “not at all,” “some” and “considerably” to “very much.” Mental disorder symptoms at the age of 31 were screened by using HSCL-25, which was included in the follow-up survey of the NFBC1966 (Veijola et al., 2003). Based on the responses, an average score was calculated, ranging from 1.0 to 4.0. A mean score of ⩾1.55 was used as a screening cut-off for having mental disorder symptoms (Koskelo et al., 2023; Sandanger et al., 1998; Veijola et al., 2003). The cut-point of 1.55 was selected due to its previously demonstrated high sensitivity in the NFBC1966 (Veijola et al., 2003). The cut-off has moderate specificity (Veijola et al., 2003), but it was compromised because we wanted to identify all individuals experiencing at least mild-to-moderate emotional distress or at risk of clinical depression or anxiety disorders.

#### Detecting Individuals with a Registered History of Mental Disorder

Psychiatric diagnoses of NFBC1966 members were retrieved from multiple national registers: the Care Register for Health Care (CRHC) (1974–) (Finnish Institute for Health and Welfare, 2024), the Register of Primary Health Care Visits (2011–) (Finnish Institute for Health and Welfare, 2024), the Social Insurance Institution of Finland (SII) (1974–) (The Social Insurance Institution of Finland, 2024), and the Finnish Centre for Pensions (FCP) (1974–) (Finnish Centre for Pensions, 2024). Individuals with any mental disorder (ICD-8: 290–308, 7,092; ICD-9: 290–316; ICD-10: F00–F99) based on the different versions of the International Classification of Diseases until 2020 were identified.

Illness onset age, meaning the age of the first occurrence of any mental disorder, was defined by using the CRHC, the register of the FCP, the SII registers of reimbursable medicines and Finnish outpatient registers.

#### Categorization of the Study Groups

To observe the effect of mental disorder symptoms and diagnoses on labour market attachment and income, we divided the sample into four sex-specific classes as follows: (1) individuals with neither self-reported symptoms nor a registered history of a mental disorder diagnosis (reference group), (2) those with symptomatic undiagnosed mental disorder, (3) those with asymptomatic diagnosed mental disorder, and (4) those with symptomatic diagnosed mental disorder.

#### Labour Market Attachment Between Ages 16 and 45

To describe labour market attachment, we used the employment trajectories previously identified in the NFBC1966 as a part of the 46-year follow-up study (Ek et al., 2021), based on a latent class analysis of all possible employment-related roles each year from 1982 to 2011. As sex-specific trajectories had been expected (McMunn et al., 2015), the latent class analysis had been performed separately by sex. Five-class solutions best fit the data for both males and females, with meaningful profiles for the identified trajectories and with slight differences by sex.

We dichotomised labour market attachment as good for those belonging to the following four employment trajectories among males and females: (1) traditional full-time employees, (2) highly educated employees, (3) self-employed, (4) delayed full-time employees. In contrast, labour market attachment was considered poor for those in the trajectories of (5) floundering employees (Ek et al., 2021). These individuals typically had a low level of education with university and applied university degrees being relatively rare and only basic or secondary education commonly completed. However, males with poor labour market attachment were clearly less educated than females. After a floundering career characterised by a high likelihood of part-time work and unemployment between ages 16 and 45, only a quarter of these individuals were in permanent full-time employment at the age of 46. Compared to individuals with good labour market attachment, unemployment and disability pensions at the age of 46 were significantly more common for those with poor labour market attachment.

#### Income Until Age 50

The information on cohort members’ taxable income was drawn from the register of the Finnish Tax Administration (until 2016) (The Finnish Tax Administration, 2024) and presented in 2016 euros. The variable included annual wage and salary earnings, self-employment earnings, and social income transfers such as sick leave benefits.

#### Background Characteristics

The following variables were used to describe the characteristics of the sample. The same variables were used as covariates in the adjusted analyses.

Information on sex (biological attribute) was retrieved from the national population register. Information from the 14-year NFBC1966 follow-up survey was used to describe the socioeconomic situation of participants’ childhood families. This was based on fathers’ socioeconomic status (SES), which was classified as either white collar or not. Data on the subjects’ average school grades (range 4–10) at the age of 16 years was gathered from the register of the Finnish national application system. Information on the highest attained educational level was retrieved from the 46-year NFBC1966 follow-up survey and classified using the International Standard Classification of Education (International Standard Classification of Education, 2011). Survey responses on marital status at the age of 46 were used and dichotomised as: (1) single, divorced, separated, or widowed and (2) married, registered partnership or cohabiting. Register data on SES at the age of 46 years was used (Statistics Finland, 2024). SES included the following categories: farmers, entrepreneurs, upper white collar, lower white collar, manual workers, students, pensioners, and others, mostly unemployed and was classified as (1) white collar that is lower to upper white collar, (2) pensioners, and (3) other categories.

### Statistical Analyses

#### Background Characteristics

The background variables in the four groups based on self-reported symptoms and mental disorder diagnoses were calculated separately by sex using cross-tabulation (categorical variables) and medians with interquartile ranges (continuous variables).

#### Labour Market Attachment

The numbers of individuals in the study groups in relation to all five employment trajectories were calculated using cross-tabulation and presented by sex.

We used logistic regression to examine the risk of poor labour market attachment, using individuals with neither mental disorder symptoms nor diagnoses as a reference category. First, we conducted an unadjusted logistic regression. Then, we adjusted the regression for the father’s SES at 14 years, individuals’ average school grades at 16 years, as well as educational level, marital status, and SES at 46 years. The results are presented as odds ratios (ORs) with 95% confidence intervals (CIs).

#### Income

The cumulative income in 1997 to 2016 between study groups was calculated separately by sex using medians with interquartile ranges and presented in 2016 euros.

Next, we used linear regression (ordinary least squares) to explore participants’ logarithmized cumulative income in 1997 to 2016. We conducted unadjusted and adjusted linear regressions similarly to when analysing labour market attachment. The results are presented as percentages (%) with 95% CIs.

#### Sensitivity Analysis

Finally, to explore the stability of self-reported symptoms in relation to income, sensitivity analysis was conducted (Online Resource 2).

All tests were two-tailed. The statistical analyses were conducted using Stata version 16.0.

## Results

### Characteristics of the Study Population and the Study Groups

Among males, 72% were in the reference group, 9% had symptomatic undiagnosed mental disorder, 14% had asymptomatic diagnosed mental disorder, and 6% had symptomatic diagnosed mental disorder (Table 1). Corresponding rates for females were 60%, 13%, 18%, and 8%, respectively.

**Table 1.** Characteristics of the Sample (n = 5,831).

Variable	Male (n = 2,600)				Female (n = 3,231)			
	Symptoms −Diagnosis −(n = 1,871)	Symptoms +Diagnosis −(n = 225)	Symptoms −Diagnosis +(n = 353)	Symptoms +Diagnosis +(n = 151)	Symptoms +Diagnosis −(n = 1,950)	Symptoms +Diagnosis −(n = 420)	Symptoms −Diagnosis +(n = 596)	Symptoms +Diagnosis +(n = 265)
Father’s SES at age 14, *n* (%)								
White collar	598 (33.9)	74 (35.6)	103 (30.9)	38 (27.7)	547 (29.5)	115 (29.6)	197 (34.5)	72 (30.4)
Other	1,168 (66.1)	134 (64.4)	230 (69.1)	99 (72.3)	1,308 (70.5)	273 (70.4)	374 (65.5)	165 (69.6)
Educational level by age 46, *n* (%)								
Basic	82 (4.4)	12 (5.4)	31 (9.0)	21 (14.4)	47 (2.4)	12 (2.9)	15 (2.5)	15 (5.7)
Secondary	894 (48.3)	121 (54.5)	198 (57.2)	89 (61.0)	722 (37.1)	165 (39.7)	252 (42.7)	124 (47.0)
Tertiary	874 (47.3)	89 (40.1)	117 (33.8)	36 (24.6)	1,178 (60.5)	239 (57.4)	323 (54.8)	125 (47.3)
Marital status at age 46, *n* (%)								
Married/registered/cohabiting	1,529 (83.4)	163 (75.1)	250 (72.0)	74 (50.7)	1,565 (81.3)	299 (72.7)	438 (74.2)	166 (64.1)
Single/divorced/separated/widowed	304 (16.6)	54 (24.9)	97 (28.0)	72 (49.3)	361 (18.7)	112 (27.3)	152 (25.8)	93 (35.9)
Socioeconomic status at age 46, *n* (%)								
White collar	880 (47.2)	86 (38.2)	128 (36.3)	33 (22.0)	1,453 (74.8)	264 (62.9)	411 (69.0)	141 (53.4)
Pensioner	15 (0.8)	6 (2.7)	21 (6.0)	26 (17.3)	15 (0.8)	5 (1.2)	31 (5.2)	39 (14.8)
Other	970 (52.0)	133 (59.1)	203 (57.7)	91 (60.7)	474 (24.4)	151 (35.9)	154 (25.8)	84 (31.8)
Average school grades at age 16, Md (IQR)	7.2 (6.5–8.1)	7.3 (6.5–8.1)	6.9 (6.2–7.6)	6.6 (5.9–7.3)	7.9 (7.1–8.7)	7.7 (6.8–8.4)	7.8 (6.9–8.6)	7.7 (6.9–8.5)
Age at onset of mental disorder, Md (IQR)			41.1 (28.6–46.0)	32.1 (27.6–41.5)			40.7 (33.3–45.5)	33.7 (28.2–37.9)
HSCL-25 score at the age 31, Md (IQR)	1.16 (1.08–1.28)	1.72 (1.60–1.84)	1.20 (1.08–1.36)	1.80 (1.68–2.08)	1.20 (1.12–1.32)	1.72 (1.60–1.88)	1.24 (1.16–1.40)	1.80 (1.64–2.04)

*Note.* SES = socioeconomic status; Md = median; IQR = interquartile range; HSCL-25 = The Hopkins Symptom Checklist-25.

Compared to other groups, individuals with symptomatic diagnosed mental disorders were more often singles and pensioners and those in the reference group had higher educational levels. In all groups, females had higher average school grades and educational levels and they were more often white-collar workers.

### Labour Market Attachment

In the reference group, 7% of males and 17% of females presented with poor labour market attachment (Table 2). Corresponding rates for poor labour market attachment were 17% for males and 18% for females with symptomatic undiagnosed mental disorder, 17% for males and 27% for females with asymptomatic diagnosed mental disorder and 36% for both males and females with symptomatic diagnosed mental disorder.

**Table 2.** Labour Market Attachment and Income in Relation to Mental Disorder Symptoms and Diagnoses.

Variable	Male (n = 2,600)				Female (n = 3,231)			
	Symptoms −Diagnosis −(n = 1,871)	Symptoms +Diagnosis −(n = 225)	Symptoms −Diagnosis +(n = 353)	Symptoms +Diagnosis +(n = 151)	Symptoms +Diagnosis −(n = 1,950)	Symptoms +Diagnosis −(n = 420)	Symptoms −Diagnosis +(n = 596)	Symptoms +Diagnosis +(n = 265)
Labour market attachment, *n* (%)								
Good	1,734 (92.7)	186 (82.7)	292 (82.7)	96 (63.6)	1,611 (82.6)	345 (82.1)	438 (73.5)	171 (64.5)
Poor	137 (7.3)	39 (17.3)	61 (17.3)	55 (36.4)	339 (17.4)	75 (17.9)	158 (26.5)	94 (35.5)
Income, Md (IQR)								
Cumulative income (€) 1997–2016	682,760 (520,540–896,700)	569,150 (404,190–760,910)	552,530 (400,250–745,020)	442,520 (220,940–588,040)	514,880 (408,640–644,150)	477,760 (359,730–605,140)	466,370 (344,200–598,590)	407,030 (265,560–509,860)
Cumulative income (€) 2012–2016	189,670 (140,200–257,670)	167,060 (108,990–222,370)	144,740 (95,060–213,080)	113,790 (44,040–177,550)	153,230 (120,470–195,600)	139,150 (104,900–177,510)	137,810 (94,060–179,840)	119,280 (54,670–163,510)

*Note*. Md = median; IQR = interquartile range.

Compared to the reference group, males but not females with symptomatic undiagnosed mental disorder had an increased risk for poor labour market attachment (OR (95% CI) 2.26 [1.41, 3.63] for males, and 0.87 [0.63, 1.19] for females) (Table 3). Having an asymptomatic diagnosed mental disorder was associated with a risk for poor labour market attachment in both sexes (OR (95% CI) 1.62 [1.05, 2.49] for males, and 1.40 [1.10, 1.79] for females). Individuals with symptomatic diagnosed mental disorders had the highest risk for poor labour market attachment in both males (OR (95% CI) 2.88 [1.66, 5.01]), and females (OR (95% CI) 1.59 [1.12, 2.25]).

**Table 3.** Odds Ratios for Poor Labour Market Attachment in Relation to Mental Disorder Symptoms and Diagnoses.

Odds ratio	Male			
	OR [95% CI]	OR [95% CI]	OR [95% CI]	OR [95% CI]
	Symptoms −Diagnosis −	Symptoms +Diagnosis −	Symptoms −Diagnosis +	Symptoms +Diagnosis +
Unadjusted	1	2.65 [1.80, 3.91]	2.64 [1.80, 3.91]	7.25 [4.99, 10.54]
Fully adjusted^a^	1	2.26 [1.41, 3.63]	1.62 [1.05, 2.49]	2.88 [1.66, 5.01]
	Female			
	OR [95% CI]	OR [95% CI]	OR [95% CI]	OR [95% CI]
	Symptoms −Diagnosis −	Symptoms +Diagnosis −	Symptoms −Diagnosis +	Symptoms +Diagnosis +
Unadjusted	1	1.03 [0.78, 1.36]	1.71 [1.38, 2.13]	2.61 [1.98, 3.45]
Fully adjusted^a^	1	0.87 [0.63, 1.19]	1.40 [1.10, 1.79]	1.59 [1.12, 2.25]

*Note.* OR = odds ratio; CI = confidence interval; SES = socioeconomic status.

a Adjusted for father’s SES at 14 years, individuals’ average school grades at 16 years, educational level, marital status at 46 years, and SES at 46 years.

### Income

The median cumulative income in 1997 to 2016 was highest for those in the reference group (682,760€ for males and 514,880€ for females) and lowest for those with symptomatic diagnosed mental disorder (442.520€ for males and 407,030€ for females) (Table 2). Individuals with symptomatic undiagnosed mental disorders (569,150€ for males and 477,760€ for females) had similar cumulative income compared to those with asymptomatic diagnosed mental disorders (552,530€ for males and 466,370€ for females). The cumulative income in 2012 to 2016 followed a similar trend.

Compared to the reference group, males with symptomatic diagnosed mental disorders had 25.5% (32.8%–17.4%) and females with symptomatic diagnosed mental disorders had 13.3% (18.1%–8.33%) lower cumulative income in 1997 to 2016 (Table 4). The cumulative income among individuals with symptomatic undiagnosed mental disorder was 14.0% (19.4%–8.15%) lower for males and 6.1% (10.4%–1.59%) lower for females, and among individuals with asymptomatic diagnosed mental disorder 14.6% (19.2%–9.79%) lower for males and 9.79% (13.1%–6.39%) lower for females compared to the reference group.

**Table 4.** Differences (in Percentage Terms) in Cumulative Income Between 1997 and 2016 in Relation to Mental Disorder Symptoms and Diagnoses.

Difference	Male			
	% [95% CI]	% [95% CI]	% [95% CI]	% [95% CI]
	Symptoms −Diagnosis −	Symptoms +Diagnosis −	Symptoms −Diagnosis +	Symptoms +Diagnosis +
Unadjusted	1	−18.13 [−24.04, −11.84]	−23.97 [−28.68, −18.86]	−47.17 [−53.51, −40.01]
Fully adjusted^a^	1	−14.02 [−19.43, −8.15]	−14.62 [−19.18, −9.79]	−25.47 [−32.77, −17.39]
	Female			
	% [95% CI]	% [95% CI]	% [95% CI]	% [95% CI]
	Symptoms −Diagnosis −	Symptoms +Diagnosis −	Symptoms −Diagnosis +	Symptoms +Diagnosis +
Unadjusted	1	−11.40 [−15.97, −6.57]	−14.96 [−18.70, −11.04]	−28.97 [−34.03, −23.51]
Fully adjusted^a^	1	−6.11 [−10.42, −1.59]	−9.79 [−13.06, −6.39]	−13.32 [−18.13, −8.33]

*Note.* CI = confidence interval; SES = socioeconomic status.

a Adjusted for father’s SES at 14 years, individuals’ average school grades at 16 years, educational level, marital status at 46 years, and SES at 46 years.

### Sensitivity Analysis

The results of the sensitivity analyses are presented in Table 5 and Online Resource 2.

**Table 5.** Differences (in Percentage Terms) in Cumulative Income Between 2012 and 2016 in Relation to Mental Disorder Symptoms at the Ages of 31 and 46.

Difference	Male			
	% [95% CI]	% [95% CI]	% [95% CI]	% [95% CI]
	Symptoms at age 31 −Symptoms at age 46 −	Symptoms at age 31 +Symptoms at age 46 −	Symptoms at age 31 −Symptoms at age 46 +	Symptoms at age 31 +Symptoms at age 46 +
Adjusted^a^	1	−14.27 [−28.39, 2.53]	−12.89 [−27.46, 4.60]	−36.24 [−51.71, −15.80]
Fully adjusted^b^	1	−10.55 [−25.92, 7.25]	−7.13 [−23.13, 12.19]	−31.68 [−48.26, −9.79]
	Female			
	% [95% CI]	% [95% CI]	% [95% CI]	% [95% CI]
	Symptoms at age 31 −Symptoms at age 46 −	Symptoms at age 31 +Symptoms at age 46 −	Symptoms at age 31 −Symptoms at age 46 +	Symptoms at age 31 +Symptoms at age 46 +
Adjusted^a^	1	−15.80 [−27.53, −2.18]	−5.07 [−14.87, 5.87]	−20.94 [−34.16, −5.07]
Fully adjusted^b^	1	−15.28 [−27.16, −1.48]	−3.76 [−13.85, 7.51]	−19.43 [−33.03, −3.08]

*Note.* CI = confidence interval; SES = socioeconomic status.

a Adjusted for father’s SES at 14 years, individuals’ average school grades at 16 years, educational level, marital status at 46 years, and SES at 46 years.

b Adjusted for father’s SES at 14 years, individuals’ average school grades at 16 years, educational level, marital status at 46 years, SES at 46 years, and for having any mental disorder diagnosis.

## Discussion

### Main Findings

To our knowledge, this is the first study comparing long-term labour market attachment and cumulative income among individuals with different histories of mental disorder symptoms and diagnoses. The study presents sex-specific differences showing that males with symptomatic undiagnosed mental disorders are at a high risk for poor labour market attachment. Females with symptomatic undiagnosed mental disorders did not have an increased risk for poor labour market attachment compared to the reference group. Compared to the reference group, having mental disorder symptoms, a diagnosis, or both was associated with lower cumulative income irrespective of sex.

### Comparison to Previous Studies

Both questionnaire-based symptoms or diagnoses and register-based diagnoses have been used to explore associations between mental health and occupational outcomes, both methods including certain advantages and disadvantages (Hakulinen et al., 2019). Our findings on labour market attachment are in line with previous Nordic studies linking mental disorder symptoms (Hiilamo et al., 2019; Pedersen et al., 2016) and diagnoses (Hakulinen et al., 2020; Majuri et al., 2023) with unfavourable occupational trajectories. A Danish study found that after a sickness absence, individuals with self-reported mental health problems were likely to spend the following 51 weeks on sickness absence and temporary disability benefits and had a high probability of not having returned to work (Pedersen et al., 2016). A Finnish study reported that individuals with self-reported common mental disorders were associated with belonging to a trajectory leading to an early exit from employment and a stable/low work disability trajectory during the 10-year follow-up period (Hiilamo et al., 2019). A Swedish study found that individuals with register-based mental disorders were likely to be in trajectories characterised by constant high levels of work disability and unemployment (Helgesson et al., 2018). Another Finnish study on employment and earnings trajectories showed that having a register-based major depressive disorder diagnosis was linked with a substantial long-term reduction in employment and earnings up to 10 years of follow-up (Hakulinen et al., 2020)). A high risk for poor labour market attachment has previously been found in the NFBC1966 among individuals with psychotic disorders, especially among males (Majuri et al., 2023). Regardless of sex and symptoms, having a mental disorder diagnosis was associated with poor labour market attachment in this study. However, we found that only males with symptomatic undiagnosed mental disorders were linked with presenting poor labour market attachment. Sex-specific differences exist between males and females with poor labour market attachment, males being less educated, which may partly explain the differences found in the study.

In terms of income, having a symptomatic diagnosed mental disorder was linked with the lowest cumulative income. Previous studies have linked both mental disorder diagnoses (Hakulinen et al., 2019) and symptoms (Jirapramukpitak et al., 2018) with a substantial loss of earnings over the life course. Irrespective of sex, having a mental disorder diagnosis was linked with lower cumulative income than having only mental disorder symptoms. This finding is in line with a previous study reporting that a more severe form of mental health disorder is associated with greater income loss (Jirapramukpitak et al., 2018). However, conclusions related to income between the diagnostic groups in our study must be interpreted carefully. Regardless of symptoms, certain disability benefits such as sickness allowances in Finland are mostly based on diagnoses which may in some cases lead to overdiagnosis in case to ensure a sufficient livelihood (Zimmerman et al., 2010). However, our findings are derived from a Nordic welfare country which provides access to social security for all citizens (Majuri et al., 2024) and which may be prone to welfare traps in cases where individuals can reach sufficient income also without disability-based benefits. It should be noted that in our study, the income variation among individuals with mental disorders was significant, reflecting the fact that mental disorders are highly diverse, their impacts on working life are different, and they occur across all income levels. In all groups, males were at a higher risk for loss of income compared to females. Following a common mental disorder diagnosis, compared to females, males have been reported to have higher odds of non-employment and sick leave (Jarl et al., 2020), which may lead to higher loss of income found in our study.

Having self-reported symptoms but being without a mental disorder diagnosis may indicate being outside the reach of services and having a potential undiagnosed mental disorder (Caspi et al., 2020; Fisher et al., 2021; Sacco et al., 2024; Young et al., 2020). Sex-specific differences in the prevalence of undiagnosed mental disorders have previously been identified (Sacco et al., 2024), undiagnosed anxiety disorders being more common among males (Fisher et al., 2021) and undiagnosed ADHD among females (Young et al., 2020). Non-help-seeking behaviour has been found particularly among males (Brandstetter et al., 2017; Oliver et al., 2005). The low levels of service utilization have been linked the with the worse recognition of mental disorder symptoms and knowledge of appropriate treatments as well as perception of stigma as a barrier to help-seeking among males (Harris et al., 2015). It has been argued that social norms of masculinity may make help-seeking more difficult for males due to inhibited emotional expressiveness, which affects symptom perception (Möller-Leimkühler, 2002). Due to non-help-seeking behaviour, males are exposed to the adverse effects of undiagnosed and untreated mental disorders. Generally, our results align with previous findings demonstrating that undiagnosed mental disorder may lead to problems related to insufficient treatment and interventions which may complicate entering and staying in the labour force (Waserstein et al., 2019).

Globally, the labour force participation rate of females is lower than that of males (International Labour Office, 2017). However, males with undiagnosed mental disorders may be exposed to a broad range of stigmatizing social conditions, such as homelessness, unemployment, incarceration and institutionalization, that affect social exclusion and labour market attachment (Williams, 2008). Generally, prevalences and spectra of mental disorders somewhat differ by sex, females having more anxiety-mood disorders than males and males having more substance disorders than females (Seedat et al., 2009). The sex-specific differences in labour market attachment may be partly related to females having more mild mental disorders such as mild depression which is not usually associated with lowered work functioning whereas males may have more severe mental disorders such as substance use disorders which may remain undiagnosed, be related to adverse social conditions, and reduce functioning (Seedat et al., 2009).

In sensitivity analysis, we studied cumulative income between 2012 and 2016 to assess the effects of symptom persistence from the age of 31 to the age of 46. As expected, individuals with symptoms at both follow-ups were linked with the highest loss in income, reflecting presumably poorer functioning and a more severe clinical presentation of mental disorders. Compared to asymptomatic individuals, among individuals with symptoms only at the age of 31 or 46, only females with symptoms at the age of 31 were associated with lower income in both adjusted models. First, this finding may reflect the long-term effects of previous symptoms in relation to income among females, even in case the health care services are reached, and treatment and other interventions are organised. Second, sex differences in the labour market exist throughout the world (International Labour Office, 2017) and this finding may reflect the unequal opportunities in the labour market between males and females with previous mental disorders or related symptoms. Compared to males, females with mental disorders have been reported to experience considerably higher levels of stigma and discrimination in some studies (Khan et al., 2015).

### Strengths and Limitations

The major strength of the study was to combine multiple register data to the birth cohort sample with longitudinal survey data on 30 years of working life. Previous studies on long-term patterns of occupational functioning and cumulative income among individuals with mental disorders tend to be cross-sectional or cover only a part of working life. Using data from multiple registers enabled us to broad data coverage with the possibility of minimising the number of potential misdiagnoses. By detecting individuals with different histories of mental disorder symptoms and register-based diagnoses, our study offers a comprehensive view of the long-term labour market attachment and income accumulation in a general population sample including individuals with diagnosed, undiagnosed, subclinical, and other states of mental disorders. Our approach enabled us to identify potential sex-specific disadvantages and advantages in career development patterns related to previous histories of mental disorders.

The study has some limitations. We included individuals with any mental disorder diagnoses until 2020 but did not pay attention to the specific timing of the diagnosis. Due to the sample size, we were not able to focus on specific categories of mental disorders such as mood disorders and psychotic disorders, which have significant differences in the course and outcome of the disorders. In addition, some of the confidence intervals were wide, particularly in relation to cumulative income, which might reflect the inherently large variation in income within the study groups, detected in a general population representing a wide range of economic sectors and occupations. Further research with larger samples in different countries remains needed.

We excluded individuals with data missing from the HSCL-25 questionnaire at age 31 or from labour market attachment at age 46. In questionnaire-based research, individuals with severe mental disorders tend to cumulate in the group of non-participants, causing selection bias (Haapea et al., 2007). Attrition analyses in our cohort have shown that non-participants in the 46-year follow-up were more often of lower socioeconomic status (Nordström et al., 2022). Compared to participants, non-participants with schizophrenia but also without a mental disorder diagnosis have been found to have worse occupational outcomes in terms of employment and education in a previous attrition analysis conducted using the same data (Majuri et al., 2023), which could affect our results. Drawing from these findings, our results may be underestimates of the associations. Moreover, the HSCL-25 is designed to identify mental disorders in primary care, and it might not be as good a measure for identifying severe psychiatric disorders treated in specialised outpatient care and psychiatric hospitals. Although we adjusted for several confounding factors from childhood into adulthood, we did not account for all potential covariates such as somatic comorbidity (Gili et al., 2010) and social support (van Es et al., 2023), which may affect the outcomes. Lastly, the registers quite comprehensively detect severe psychiatric disorders but have not detected all milder disorders; for example, those diagnosed and treated solely in occupational health care which has gradually joined the primary care registers only since 2019, or disorders that have not been diagnosed or treated within health care at all due to the non-help seeking behaviour or lack of available services. In the future, studies focusing on different categories of mental disorders and accounting for additional confounding factors are needed to draw more specific conclusions.

### Clinical Implications

From the work-life perspective, understanding the negative effects of mental disorder symptoms or diagnosis is important. For employers, mental disorders can be costly in terms of lost productivity and for individual employees, mental health issues may have negative effects not only on income, as we have shown in this study, but also on quality of life and workplace engagement (Xu et al., 2022). Recently, there has been an increasing trend in screening individuals with poor mental health in the workplace to reduce these effects (Strudwick et al., 2023). Health screening questionnaires have been developed to detect potential psychosocial distress, such as depression and anxiety, in order to organise appropriate interventions to ensure the wellbeing of the worker (Bohatko-Naismith et al., 2022). With a more contextual approach, the recent EU-level guidance on best practices to support mental health in the workplace aims to provide practical tools for local interventions and to overcome stigma and other barriers (European Union Agency for Safety and Health at Work, 2024). Our findings suggest that interventions should be considered not only based on diagnoses after the onset of mental disorders but also based on symptoms during early stages, as they may serve as predictors of future challenges related to work.

In health care practices, effective interventions like The Individual Placement and Support (IPS) approach (Bond et al., 2023) should be implemented more widely to support individuals in all stages of the process of finding and maintaining employment. From the policymaker perspective, agreements like The Inclusive Workplace Agreement (Hasting et al., 2022) between employees and authorities could be adopted in Finland to make investing in an employee with a mental disorder more beneficial from the employer’s perspective.

In Finland, outreach work is organised for individuals under the age of 29 to support these people in accessing the health and community services (The Youth Act, 2016). Regarding our findings and the non-help-seeking behaviour among certain individuals such as males with mental disorders (Brandstetter et al., 2017; Oliver et al., 2005), outreach work should be considered based not only on age but also based on not being in education, employment, and training (Ringbom et al., 2022) to detect individuals with potential undiagnosed mental disorders and improve labour market attachment among them.

## Conclusion

Mental disorder symptoms and diagnoses are differently associated with labour market attachment and registered income among males and females. Our findings suggest that interventions should be offered based not only on diagnoses but also on mental disorder symptoms, which may serve as predictors of future challenges related to working life. Males with an undiagnosed mental disorder are at a high risk for poor labour market attachment, reflecting the need for more effective interventions for these individuals characterised by non-help-seeking behaviour.

## Supplemental Material

sj-docx-1-isp-10.1177_00207640241299384 – Supplemental material for Mental disorder symptoms and diagnoses are differently associated with labour market attachment and registered income until midlife: The Northern Finland Birth Cohort 1966 Supplemental material, sj-docx-1-isp-10.1177_00207640241299384 for Mental disorder symptoms and diagnoses are differently associated with labour market attachment and registered income until midlife: The Northern Finland Birth Cohort 1966 by Tuomas Majuri, Sanna Huikari, Erika Jääskeläinen, Leena Ala-Mursula, Ina Rissanen and Marko Korhonen in International Journal of Social Psychiatry

sj-docx-2-isp-10.1177_00207640241299384 – Supplemental material for Mental disorder symptoms and diagnoses are differently associated with labour market attachment and registered income until midlife: The Northern Finland Birth Cohort 1966 Supplemental material, sj-docx-2-isp-10.1177_00207640241299384 for Mental disorder symptoms and diagnoses are differently associated with labour market attachment and registered income until midlife: The Northern Finland Birth Cohort 1966 by Tuomas Majuri, Sanna Huikari, Erika Jääskeläinen, Leena Ala-Mursula, Ina Rissanen and Marko Korhonen in International Journal of Social Psychiatry
