# Supplementary material for: Mental disorder symptoms and diagnoses are differently associated with labour market attachment and registered income until midlife: The Northern Finland Birth Cohort 1966
Source: Int J Soc Psychiatry. 2024 Nov 27;71(4):682–93. doi: 10.1177/00207640241299384 (PMC12171070; doi:10.1177/00207640241299384)
Supplement: sj-docx-1-isp-10.1177_00207640241299384 – Supplemental material for Mental disorder symptoms and diagnoses are differently associated with labour market attachment and registered income until midlife: The Northern Finland Birth Cohort 1966 [file sj-docx-1-isp-10.1177_00207640241299384.docx]

International Journal of Social Psychiatry

Mental disorder symptoms and diagnoses are differently associated with labour market attachment and registered income until midlife: the Northern Finland Birth Cohort 1966


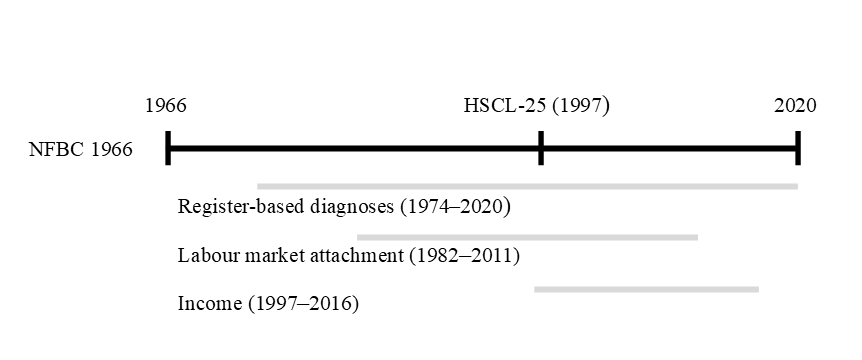
Online Resource 1.

**Fig. 1** Timeline of data collection in the study
