# Supplementary material for: Mental disorder symptoms and diagnoses are differently associated with labour market attachment and registered income until midlife: The Northern Finland Birth Cohort 1966
Source: Int J Soc Psychiatry. 2024 Nov 27;71(4):682–93. doi: 10.1177/00207640241299384 (PMC12171070; doi:10.1177/00207640241299384)
Supplement: sj-docx-2-isp-10.1177_00207640241299384 – Supplemental material for Mental disorder symptoms and diagnoses are differently associated with labour market attachment and registered income until midlife: The Northern Finland Birth Cohort 1966 [file sj-docx-2-isp-10.1177_00207640241299384.docx]

International Journal of Social Psychiatry

Mental disorder symptoms and diagnoses are differently associated with labour market attachment and registered income until midlife: the Northern Finland Birth Cohort 1966

Online Resource 2.

**Methods**

**Statistical analyses**

***Sensitivity analysis***

Finally, to explore the stability of self-reported symptoms in relation to income, we used additional data on HSCL-25 scores at the age of 46 from the NFBC1966 follow-up survey and divided the sample into four classes based on the HSCL-25 mean scores at the ages of 31 and 46. The classes were: 1) asymptomatic (HSCL-25 mean score <1.55) at the ages of 31 and 46, 2) those with symptoms only at the age of 31, 3) those with symptoms only at the age of 46, and 4) those with symptoms at both ages of 31 and 46.

In sensitivity analysis, linear regression (ordinary least squares) was used to analogously explore logarithmized cumulative income in 2012-2016 between the new study groups using individuals without symptoms at the ages of 31 and 46 as a reference category. In the first model, we adjusted the regression for the father’s SES at 14 years, average school grades at 16 years, educational level, marital status and SES at 46 years. In the second model, regression was adjusted for all these variables together with information on having any psychiatric diagnosis until 2020 based on the registers used in the case detection phase.

**Results**

**Sensitivity analysis**

Compared to asymptomatic individuals, females with symptoms at the age of 31 had significantly 15.3% (27.2%–1.48%) lower cumulative income, whereas for males the difference was not statistically significant -10.6% (-25.9%–7.25%). There were no statistically significant differences in cumulative income between asymptomatic individuals and those with symptoms at the age of 46 (differences in income were -7.13% (-23.1%–12.2%) for males and -3.76% (-13.9%–7.51%) for females, respectively). Individuals with mental disorder symptoms at the ages of 31 and 46 had significantly lower cumulative income between 2012 and 2016 compared to those who were asymptomatic (31.7% (48.3%–9.79%) for males, 19.4% (33.0%–3.08%) for females).
